# Perceptual restoration of locally time-reversed speech: Japanese words are very tolerant of severe temporal distortion

**DOI:** 10.3758/s13414-025-03114-6

**Published:** 2025-07-21

**Authors:** Mako Ishida, Takayuki Arai, Makio Kashino

**Affiliations:** 1 Faculty of Science and Technology, Department of Foreign Language and Liberal Arts, Keio University, 4 Chome-1-1 Hiyoshi, Kohoku Ward, Yokohama, Kanagawa 223-8521 Japan; 2 Sophia University, Tokyo, Japan; 3 NTT Communication Science Laboratories, Atsugi, Japan

**Keywords:** Perceptual restoration, Locally time-reversed speech, Japanese words and pseudowords

## Abstract

People can understand speech even when the speech signal is divided into equally long segments and each segment is reversed in time (locally time-reversed speech). In addition, Ishida (*Attention, Perception & Psychophysics, 83*(6), 2675–2693, 2021) reported that Japanese words – composed of consonant–vowel (CV) units – were significantly more intelligible than English words when locally time-reversed. The current study investigates how tolerant and robust Japanese words are under more severe temporal distortions. In Experiment 1, native Japanese speakers listened to Japanese words and pseudowords that were locally time-reversed at intervals of 100, 120, 140, 160, 180, and 200 ms, which had not been previously examined. These lexical items contained either many fricatives or stops. Results showed that Japanese words were highly tolerant of local time reversal, even at these extreme durations. Perceptual restoration was sustained by dominant phoneme type (fricative-dominant > stop-dominant) and lexicality (words > pseudowords). In Experiment 2, participants listened to *stop-dominant* Japanese words and pseudowords, which were more susceptible to temporal distortion in Experiment 1. Temporal distortion was further increased by introducing extreme speech rates (fast vs. slow) while reversing the signal at 10, 30, 50, 70, 90, and 110 ms, commonly used intervals with normal speech rates. Results showed that *stop-dominant* Japanese words remained intelligible with increasing distortions, while pseudowords remained intelligible only up to 50 ms in the slow condition and became unintelligible in the fast condition. Overall, recognition of Japanese CV-based words was highly tolerant of severe temporal distortion, with perceptual restoration supported by dominant phoneme type, slower speech rate, and lexicality.

## Introduction

Perceptual restoration is a type of illusion in which degraded information is perceptually restored and perceived as intact (Warren, 1970; Warren & Warren, 1970). People frequently experience perceptual restoration in daily conversations, particularly in the auditory domain. For instance, when surrounded by random noise or when a public announcement at a station is partially missing or degraded due to a malfunctioning loudspeaker, people can still comprehend the message by perceptually restoring the degraded parts of speech. The human brain can retrieve both auditory and linguistic cues, integrate them, and fill in the gaps in speech – a capability that machines cannot yet fully replicate.

**Fig. 1:**
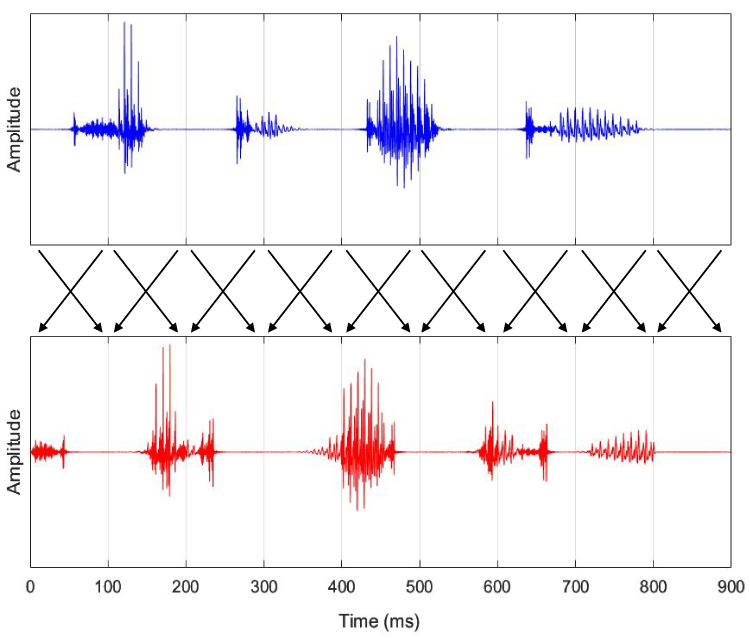
The speech signal of locally time-reversed speech. The upper panel shows the speech signal of the stop-dominant Japanese word “kakutoku” (meaning “acquire”). The lower panel shows the speech signal of the same word, locally time-reversed at 100-ms intervals. To prevent additional clicks, the edges of the reversed segments were crossfaded with a 5-ms linear amplitude ramp, following the procedures adopted by Ishida et al. (2016), Ishida et al. (2018), and Ishida (2021)

Saberi and Perrott (1999) demonstrated that listeners could understand speech even when the speech signal was quite drastically distorted, as in “locally time-reversed speech” (Fig. 1). In locally time-reversed speech, the signal is divided into equally timed segments (e.g., 100 ms) from the onset (e.g., 0–100 ms, 100–200 ms, 200–300 ms), with each segment flipped in time (e.g., 100–0–200–100–300–200). In other words, the temporal constituents of the speech signals are shifted forward or backward from their original temporal positions due to local time reversal. However, listeners can still understand speech by integrating the dispersed information over time.

This phenomenon has been supported by subsequent studies on locally time-reversed speech, primarily at the sentence level. Perceptual restoration of locally time-reversed “sentences” has been confirmed in English (Greenberg & Arai, 2001; Ishida et al., 2018; Remez et al., 2013; Stilp et al., 2010; Ueda et al., 2017), French (Magrin-Chagnolleaue et al., 2002), German (Kiss et al., 2008; Ueda et al., 2017), Mandarin Chinese (Ueda et al., 2017), and Japanese (Ishida et al., 2018; Ueda & Ciocca, 2021; Ueda et al., 2017). While Magrin-Chagnolleau et al. (2002) discussed the influence of language structure and intelligibility measurement on perceptual restoration of locally time-reversed sentences by comparing their results in French (a syllable-timed language) with those in English (a stress-timed language; as reported in Greenberg & Arai, 2001), the general trend is that the intelligibility of locally time-reversed sentences gradually deteriorates as the reversed segment length increases. Speech is typically understood at about half-intelligibility with a reversed segment length of 60–70 ms, becoming unintelligible when the reversed segment length reaches approximately 100 ms. While perceptual restoration at the sentence level can be greatly affected by sentential, lexical, and phonemic contexts (Samuel, 1981a, 1981b; Warren & Sherman, 1974; Warren & Warren, 1970) as well as linguistic proficiency (Ishida et al., 2018; Kiss et al., 2008; Warren & Warren, 1970), locally time-reversed sentences generally become unintelligible when the reversed segment length exceeds 100 ms.

**Fig. 2:**
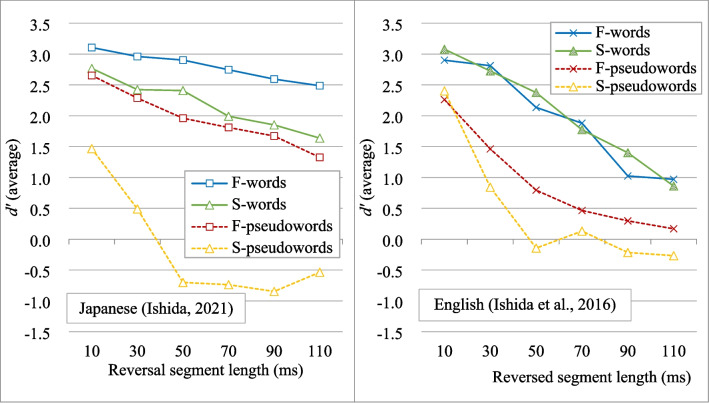
Intelligibility of locally time-reversed words and pseudowords in Japanese and English for comparison. A *d’* value lower than 0.5 generally indicates chance-level intelligibility. The Japanese data are from Ishida (2021) and the English data are from Ishida et al. (2016). F-words: fricative-dominant words, S-words: stop-dominant words, F-pseudowords: fricative-dominant pseudowords, S-pseudowords: stop-dominant pseudowords

Beyond the sentence level, perceptual restoration has also been examined in isolated words and pseudowords, demonstrating that intelligibility can be maintained even in the absence of sentential context. Studies in Japanese (Ishida, 2021) and English (Ishida et al., 2016) have shown that words and pseudowords remain intelligible when local time reversal is applied, using the same acoustic manipulations, experimental procedures, and measurements (Fig. 2). Intelligibility gradually declined as reversed segment length increased, and perceptual restoration was consistently supported by lexicality (words > pseudowords) and dominant phoneme type in the stimuli (fricative-dominant > stop-dominant) across both languages. However, intelligibility of locally time-reversed stimuli – particularly fricative-dominant words and pseudowords, as well as stop-dominant words – was generally much higher in Japanese than in English, even under the most drastic temporal distortion with a 110-ms reversed segment length. The key questions here are: (1) To what extent can Japanese words and pseudowords tolerate distortion produced by local time reversal based on rather long segment durations? (2) Does the observed robustness reflect language-specific characteristics (e.g., mora-timed Japanese vs. stress-timed English), as proposed by Magrin-Chagnolleau et al. (2002) at the sentence level? While perceptual restoration of locally time-reversed speech involves both bottom-up acoustic processing and top-down linguistic processing, past studies have focused mostly on sentence-level restoration in syllable-oriented languages such as English. There remains much room to investigate the mechanisms of perceptual restoration at a lexical level across different languages, including the roles of lexical and phonemic context as well as the effects of language-specific linguistic and acoustic structure.

The current study investigates the robustness of Japanese words and pseudowords under more severe local time distortions, highlighting the possible uniqueness of the Japanese language. It adopts the same acoustic manipulations, experimental procedures, and measurements as previous studies at the lexical level but under different experimental conditions. Experiment 1 examines the intelligibility of Japanese words and pseudowords with reversed segment lengths exceeding 100 ms in six steps (100, 120, 140, 160, 180, and 200 ms). These segment lengths were selected based on Ishida (2021), who found that Japanese words and pseudowords retained high intelligibility at 110 ms, unlike in English. This experiment tests whether lexical (words > pseudowords) and phonemic factors (fricative-dominant > stop-dominant) still contribute to perceptual restoration under severe temporal distortions beyond 100 ms – an effect not previously examined at the lexical level. Experiment 2 further investigates the intelligibility of Japanese words and pseudowords with a high number of stops under multiple temporal distortions. Stop-dominant words and pseudowords are known to be susceptible to local time distortion (Ishida, 2021; Ishida et al., 2016) and influenced by speech rate (Ishida, 2017). This experiment examines the intelligibility of stop-dominant words and pseudowords by introducing extreme speech rates (fast vs. slow; naturally controlled by the speaker), while applying local time reversal at intervals of 10, 30, 50, 70, 90, and 110 ms – commonly used in our previous studies with normal speech rates (Ishida, 2021; Ishida et al., 2016, 2018). Unlike Experiment 1, which investigates the perceptual tolerance of normally spoken Japanese words and pseudowords beyond 100 ms, Experiment 2 focuses on the effects of extreme speech rates on stop-dominant stimuli, compared to normal speech (previously examined in Ishida, 2021). Both experiments assess the robustness of Japanese words and pseudowords under previously unexamined conditions – longer reversed segment lengths and varying speech rates. Upon confirming the unique perceptual tolerance of Japanese words and pseudowords under two distinct types of severe temporal distortions, in the *Discussion* section this study discusses the potential linguistic and acoustic factors that contribute to their high intelligibility compared to English.

## Experiment 1

Experiment 1 examines how Japanese words and pseudowords tolerate local time reversal when more severe temporal distortions are applied, compared to previous studies. The study adopts local time reversal at segment lengths of 100 ms or more in six steps, reflecting previous findings that locally time-reversed Japanese words and pseudowords retained high intelligibility at 110 ms in Ishida (2021), compared to English (Ishida et al., 2016). A within-subjects design is used with three main factors: dominant-phoneme type (fricative-dominant vs. stop-dominant), lexicality (word vs. non-word), and reversed segment length (100–200 ms). Using the same acoustic manipulations, experimental procedures, and measurements as previous studies – but with longer reversed segment lengths – this experiment examines the potential linguistic and acoustic factors contributing to the high robustness of locally time-reversed Japanese words and pseudowords.

### Participants

Thirty native Japanese speakers (19 female, 11 male) participated in the experiment at NTT Communication Science Laboratories in Japan. The sample size was determined based on previous research in Japanese (Ishida, 2021) and English (Ishida et al., 2016). The average age of participants was 34.5 years. None reported any hearing or speech impairments. All collected data were used for analysis, with no exclusions.

### Materials

The set of Japanese words and pseudowords for the current experiment was adopted from Ishida (2021), to further examine the intelligibility of Japanese words and pseudowords under more severe temporal distortions. All Japanese stimuli were created following the procedures adopted for Japanese stimuli in Ishida (2021) and English stimuli in Ishida et al. (2016) for comparison.

There were 60 pairs of fricative-dominant Japanese words and pseudowords and 60 pairs of stop-dominant Japanese words and pseudowords (Appendix A). *Words* in these stimuli were originally selected from “Nihon go no goi tokusei” (Amano & Kondo, 2000), a Japanese word database. This database includes Japanese words with three “presentation-dependent” familiarity ratings: (1) orthography + sound familiarity, (2) orthography-only familiarity, and (3) sound-only familiarity. The familiarity ratings were on a 7-point scale (7: familiar, 1: unfamiliar), and all words selected for this study had familiarity ratings above 5 for all three criteria. The average familiarity rating was 5.61 for fricative-dominant words and 5.66 for stop-dominant words. Both fricative- and stop-dominant words were four morae long, with the structure CVCVCVCV (C: consonant, V: vowel). While the Japanese language allows for V as a basic mora and C or CCV as a special mora (Kono, 2004) (see Table 1), the stimuli included only the basic CV mora, the most common structure in Japanese (Kawagoe, 2007; Kono, 2004). Fricative-dominant words contained more fricatives (*M* = 2.13) than stops (*M* = 1.02), and stop-dominant words contained more stops (*M* = 3.25) than fricatives (*M* = 0.17) – this resulted in the number of fricatives in fricative-dominant words being about 12 times higher than in stop-dominant words, and the number of stops in stop-dominant words being about three times higher than that in fricative-dominant words, under the constraints of the Japanese phonotactics. After selecting fricative-dominant and stop-dominant words, matched pseudowords were created by changing the first phoneme of either the second, third, or fourth mora into another phoneme that has a different place of articulation (e.g., “ka**k**utoku” was changed to “ka**p**utoku”). The subtle difference between words and pseudowords was intentionally created for the same-different task, to use *d'* from signal detection theory as an index of intelligibility, as in previous studies (Ishida, 2021; Ishida et al., 2016).

**Table 1:** Comparison of the structure of basic sound units in Japanese (mora) and English (syllable). Concepts and examples are drawn from Bergman et al. (2007), Celce-Murcia et al. (2010) and Kono (2004); Japanese example words are original to this study

Basic mora	IPA transcription	Example word	Meaning	# of mora
CV	/ki/	木	tree	1
V	/e/	絵	picture	1
Special mora	IPA transcription	Example word	Meaning	# of mora
C	/Q/ in /kaQko/	括弧	parenthesis	3
	/N/ in /kaN/	缶	can (container)	2
CCV	/kjo/ in /kjou/	今日	today	2
Syllable	IPA transcription	Example word		
V	/ə/	a		
VC	/æt/	at		
VCC	/æsk/	ask		
VCCC	/æskt/	asked		
CV	/noʊ/	no		
CVC	/nɑt/	not		
CVCC	/ræmp/	ramp		
CVCCC	/ræmps/	ramps		
CVCCCC	/sɪksθs/	sixths		
CCV	/flu/	flew		
CCVC	/flut/	flute		
CCVCC	/fluts/	flutes		
CCVCCC	/kræfts/	crafts		
CCVCCCC	/ɡlɪmpst/	glimpsed		
CCCV	/spri/	spree		
CCCVC	/splin/	spleen		
CCCVCC	/strɛŋθ/	strength		
CCCVCCC	/strɛŋθs/	strengths		

For the recording of the Japanese stimuli, male and female native Japanese speakers read the set of fricative-dominant and stop-dominant words and pseudowords at their natural speed. The recording was conducted in a sound-shielded room at NTT Communication Science Laboratories, using a microphone (SONY ECM-MS957) and a digital audio recorder (SONY PCM-D50). The audio was recorded at a sampling rate of 44,100 Hz with 16-bit resolution and stored as a WAV file. After visually and auditorily confirming that no noise was included in the audio, the sound level was normalized based on the peak amplitude level using GoldWave, a digital audio editing software (with the Maximize Volume – Half Dynamic Range function). Sound normalization was conducted before segmenting the audio into individual words and pseudowords for stimuli creation, as in previous studies. This process ensured that the sound was loud enough for listeners to hear clearly without exceeding the maximum digital value or causing clipping artifacts, while achieving consistent loudness (by avoiding overly loud or quiet sounds) and preserving the natural transitions of speech sounds. The words and pseudowords in the male voice were then locally time-reversed at intervals of 100, 120, 140, 160, 180, or 200 ms from the onset of speech, using custom software running on MATLAB (as also adopted in Ishida, 2017, 2021; Ishida et al., 2016, 2018). To alleviate additional clicks and noise expected at the edges, the 5-ms of onset and offset of adjacent reversed segments were cross-faded using a linear ramp, consistent with procedures in previous studies.

### Procedure

The basic experimental procedure followed previous studies by Ishida (2021) and Ishida et al. (2016). The experiment was conducted in a sound-shielded room at NTT Communication Science Laboratories in Japan. There were two experimental sessions with different stimuli: (1) fricative-dominant stimuli, and (2) stop-dominant stimuli. Half of the participants started with the fricative-dominant stimuli, followed by the stop-dominant stimuli, and the other half started with the stop-dominant stimuli, followed by the fricative-dominant stimuli, for counterbalancing purposes.

In the experiment, participants sat in front of a computer and listened to stimuli through headphones (SONY MDR-CD900ST). The stimuli were presented at the participants’ comfortable listening level, and the computer and headphones were connected via a USB audio interface (Roland UA-25 EX) that served as a digital-to-analog converter. In each trial, participants heard a pair of items: a locally time-reversed item in a male voice (i.e., target stimulus), followed by a naturally spoken item in a female voice (i.e., standard stimulus). Their task was to judge whether the first and second speakers said the “same” or “different” words by pressing a button on a response pad. The trials had four possible pairs: (1) word-word for “same”; (2) word-pseudoword for “different”; (3) pseudoword-word for “different”; and (4) pseudoword-pseudoword for “same.” The gender of the first and second speakers was intentionally changed so that participants judged “same” or “different” based not on the acoustic similarity between the paired stimuli but on the Japanese word they had in mind while listening (Ishida, 2021; Ishida et al., 2016). Each participant completed 240 trials with four pairs × 60 items. The 60 items consisted of six subsets of ten items, with each subset assigned to one of the six reversed segment lengths (100–200 ms). There were six groups of participants in a Latin square design, to counterbalance the six subsets of items across six levels of reversed segment lengths. Each participant experienced 240 trials in a randomized order, with each stimulus presented with 400-ms inter-stimulus intervals. The total duration of the two experimental sessions was approximately 1 h.

## Results and discussion

The current study adopted the same–different task to examine the intelligibility of locally time-reversed lexical items using *d’* from signal detection theory, following previous studies (Ishida, 2021; Ishida et al., 2016). In this task, participants listen to a pair consisting of a locally time-reversed item (target) and a normally spoken item (standard) and judged whether the two speakers said the same or different words. A word and a matched pseudoword differed only by one phoneme (e.g., kakutoku vs. kaputoku), and this subtle difference was intentionally created to compute *d’* as a measure of intelligibility. For each participant, *d’* was computed for words and pseudowords for six levels of speech degradation (100–200 ms). When miss or false rates were at ceiling or floor, the value was replaced by either the value of 1/2N (floor) or 1–1/2N (ceiling), where N represents the number of items.

**Fig. 3:**
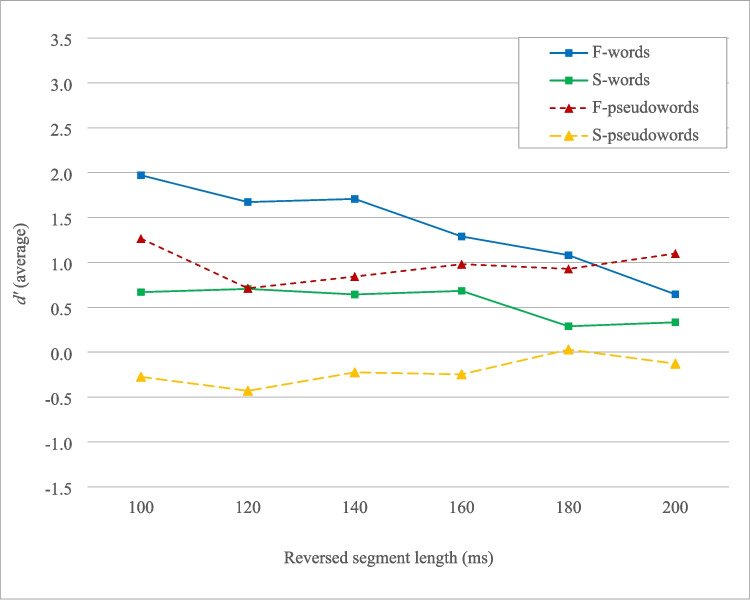
Intelligibility of locally time-reversed fricative-dominant and stop-dominant Japanese words and pseudowords, as measured in Experiment 1. A *d’* value lower than 0.5 generally indicates chance-level intelligibility. F-words: fricative-dominant words, S-words: stop-dominant words, F-pseudowords: fricative-dominant pseudowords, S-pseudowords: stop-dominant pseudowords

Figure 3 shows that locally time-reversed fricative-dominant words and pseudowords remained intelligible across six levels of speech degradation, with a *d'* = 0.5 approximately representing chance-level intelligibility (*d'* = 1.97, 1.67, 1.71, 1.29, 1.08, and 0.64 for fricative-dominant words; *d'* = 1.27, 0.71, 0.84, 0.98, 0.93, and 1.10 for fricative-dominant pseudowords). In contrast, stop-dominant words lost their intelligibility when the reversed segment length exceeded 180 ms (*d’* = 0.67, 0.71, 0.64, 0.68, 0.29, and 0.33), and stop-dominant pseudowords were largely unintelligible (*d’* = −0.27, −0.43, −0.22, −0.25, 0.03, and −0.13). Overall, fricative-dominant Japanese words and pseudowords remained intelligible, even as degradation increased with the reversed segment length from 100 to 200 ms, compared to their stop-dominant counterparts. Additionally, words were generally more intelligible than pseudowords under severe temporal distortion, although the lexical advantage (the gap between words and pseudowords) diminished when the reversed segment length exceeded 160–180 ms. Japanese words and pseudowords were generally very tolerant of local time reversal compared to English, while phonemic and lexical effects remained evident in perceptual restoration.

An ANOVA was performed with dominant phoneme type (fricative vs. stop), lexicality (word vs. pseudoword), and reversed segment length (100–200 ms) as within-subjects factors. The results showed that fricative-dominant stimuli were significantly more intelligible than stop-dominant stimuli when locally time-reversed, *F*(1, 29) = 94.92, *p* < 0.001, partial η^2^ = 0.77. Words were also significantly more intelligible than pseudowords when locally time-reversed, *F*(1, 29) = 18.29, *p* < 0.001, partial η^2^ = 0.39. In addition, the intelligibility of locally time-reversed stimuli significantly deteriorated with increasing reversed segment length, *F*(5, 145) = 7.75, *p* < 0.001, partial η^2^ = 0.21. There was also a significant interaction between dominant phoneme type and lexicality, *F*(1, 29) = 5.83, *p* = 0.02, partial η^2^ = 0.17, suggesting that the intelligibility gap between words and pseudowords was significantly smaller for fricative-dominant stimuli than for stop-dominant stimuli. Furthermore, there was a significant interaction between dominant phoneme type and reversed segment length, *F*(4.61, 133.73) = 5.02, *p* < 0.001, partial η^2^ = 0.15 (Huynh–Feldt corrections), indicating that the intelligibility degradation pattern of fricative-dominant stimuli with increasing reversed segment length differed significantly from that of stop-dominant stimuli. Additionally, there was a significant interaction between lexicality and reversed segment length, *F*(5, 145) = 13.39, *p* < 0.001, partial η^2^ = 0.32, suggesting that the intelligibility deterioration pattern of words with increasing reversed segment length, was significantly different from that of pseudoword. Overall, there was a small but significant three-way interaction among the dominant phoneme type, lexicality, and reversed segment length, *F*(5, 145) = 2.31, *p* = 0.047, partial *η*^2^ = 0.07. This indicates that the intelligibility gap between fricative-dominant words and pseudowords transitioned significantly differently as the reversed segment length increased, compared to the intelligibility gap between stop-dominant words and pseudowords.

Altogether, Japanese lexical items were generally very tolerant of local time distortion – they remained intelligible even when local time reversal was imposed at intervals of 100 ms or more, a condition not previously examined at the lexical level (Ishida, 2021; Ishida et al., 2016, 2018). Fricative-dominant Japanese stimuli were more intelligible than stop-dominant Japanese stimuli, and fricative-dominant Japanese *pseudowords* were even more intelligible than stop-dominant Japanese *words*. The strong robustness of fricative-dominant Japanese stimuli may be attributed to the acoustic characteristics of fricatives, which have a relatively symmetric waveform that remains largely intact even when locally time-reversed. In contrast, stops have an asymmetric acoustic structure, with voice onset time (VOT) that can be more severely impaired when locally time-reversed. Even with lexical support (words > pseudowords), fricative-dominant Japanese pseudowords were more intelligible than stop-dominant Japanese words, reflecting the robustness of fricative-dominant stimuli to temporal distortions. Overall, Japanese words and pseudowords were generally much more intelligible than their English counterparts, supported by dominant phoneme type (fricative > stop) and lexicality (words > pseudowords), consistent with previous studies at the lexical level (Ishida, 2021; Ishida et al., 2016).

## Experiment 2

Building on Experiment 1, where stop-dominant Japanese words and pseudowords were highly susceptible to severe temporal distortions (despite the general robustness of Japanese stimuli), Experiment 2 further examines the intelligibility of locally time-reversed stop-dominant stimuli across varying speech rates. Specifically, Experiment 2 examines how intelligible stop-dominant Japanese words and pseudowords can be when spoken at a “fast” or “slow” speech rate, compared to those spoken at a normal speech rate (Ishida, 2021; Ishida et al., 2016). In general, “fast speech” is expected to be more challenging to understand because speakers tend to move their articulators (e.g., tongues, teeth, lips, and jaws) quickly, which can result in the omission or alteration of speech sounds (Brown & Hilferty, 1982, 1986, 1995; Brown & Kondo-Brown, 2006; Dalby, 1986; Johnson, 2004). Conversely, “slow speech” is expected to be more intelligible as speakers tend to pronounce phonemes and words more clearly. Experiment 2 introduced extreme speech rates (fast vs. slow) while adopting local time reversals at 10–110 ms – intervals commonly used at normal speech rates in our previous studies (Ishida, 2021; Ishida et al., 2016, 2018) – in order to examine the effects of extreme speech rate on the intelligibility of locally time-reversed speech. Experiment 2 uses a within-subjects design with three main factors: speech rate (fast vs. slow), lexicality (word vs. non-word), and reversed segment length (10–110 ms). Following the same acoustic manipulations, experimental procedures, and measurements as previous studies, this experiment explores the robustness of Japanese stop-dominant stimuli under changes in speech rate controlled naturally by a speaker.

### Participants

Thirty native Japanese speakers (19 female, 11 male) were newly recruited for the experiment at NTT Communication Science Laboratories in Japan. The average age of the participants was 37.3 years, and none reported any hearing or speech impairments. As in Experiment 1, the sample size was determined based on previous studies (Ishida, 2021; Ishida et al., 2016). All collected data were used for analysis, with no exclusions.

### Materials

The target stimuli for Experiment 2, which were to be locally time-reversed, were newly prepared using the same set of 60 stop-dominant Japanese words and pseudowords from Experiment 1. These were then re-recorded at either a fast or slow speech rate, naturally controlled by the speaker. The speech rate was adjusted by the speaker, not by machines or software, to examine how people understand speech at varying speech rates that may occur in natural settings. The speaker was the same male individual who pronounced the stop-dominant Japanese words and pseudowords at a normal speech rate in Experiment 1. For the “fast” stimuli, the speaker pronounced the words and pseudowords as quickly as possible. An independent *t*-test showed that the duration of fast-spoken lexical items (*M* = 0.64, *SD* = 0.07) was significantly shorter than that of normally spoken lexical items (*M* = 0.92, *SD* = 0.06) from Ishida (2021), *t* (238) = 33.48, *p* < 0.001, 95% CI [0.26 0.29], *d* = 4.32. For the “slow” stimuli, the same speaker pronounced the words and pseudowords as slowly as possible. Another independent *t*-test confirmed that the duration of slowly spoken lexical items (*M* = 1.78, *SD* = 0.07) was significantly longer than that of normally spoken items (*M* = 0.92, *SD* = 0.06) from Ishida (2021), *t* (238) = −99.79, *p* < 0.001, 95% CI [−0.88 −0.84], *d* = 12.89. The duration of the fast- and slow-spoken stimuli differed significantly from that of their normal counterparts.

As in Experiment 1, the recording was conducted at NTT Communication Science Laboratories using the same apparatus and procedures. The recorded audio was stored as WAV files at a sampling rate of 44,100 Hz with 16-bit resolution. Sound levels were normalized using GoldWave. After normalization, the individual words and pseudowords were segmented from the audio, and then locally time-reversed at intervals of 10, 30, 50, 70, 90, or 110 ms using MATLAB.

### Procedure

The experiment was conducted at NTT Communication Science Laboratories using the same apparatus and procedures as in Experiment 1. There were two experimental sessions with different stimuli: (1) fast stimuli and (2) slow stimuli. Half of the participants started with the fast stimuli followed by the slow stimuli, and the other half started with the slow stimuli, followed by the fast stimuli, for counterbalancing purposes.

In the experiment, participants listened to a locally time-reversed item in a male voice (target; spoken either at a fast or slow tempo), followed by a naturally spoken item in a female voice (standard; spoken at a normal tempo). They then judged whether the first and second items were “same” or “different” by pressing a button on a response pad. There were four possible pairs: (1) word-word (same), (2) word-pseudoword (different), (3) pseudoword-word (different), and (4) pseudoword-pseudoword (same). Each participant experienced 240 trials (four pairs × 60 items). The 60 items consisted of six subsets of ten items, each assigned to one of the six reversed segment lengths (10–110 ms). Six groups of participants counterbalanced the six subsets of items across six levels of reversed segment lengths. The inter-stimulus interval was 400 ms, and the total duration of the two experimental sessions was approximately 1 h.

### Results and discussion

The current study examined the intelligibility of locally time-reversed stop-dominant Japanese words and pseudowords spoken at “fast” and “slow” speech rates using the same–different task and computing *d’* from signal detection theory, compared to those spoken at a “normal” speech rate as reported in Ishida (2021).

**Fig. 4:**
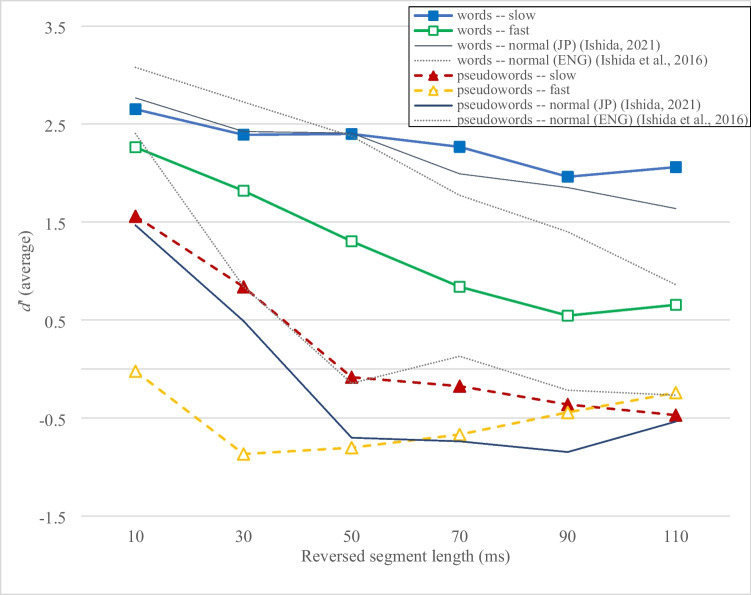
Intelligibility of locally time-reversed stop-dominant Japanese words and pseudowords spoken at “fast” and “slow” tempos in Experiment 2. The intelligibility of words spoken at a normal tempo in Japanese (JP) is from Ishida (2021), and in English (ENG) from Ishida et al. (2016), shown for comparison. A *d'* value lower than 0.5 generally indicates chance-level intelligibility. The speech rates were naturally controlled by the speaker

Figure 4 shows that locally time-reversed *words* were consistently more intelligible than *pseudowords* when spoken at a “slow” speech rate (*d’* = 2.65, 2.39, 2.40, 2.27, 1.96, and 2.06 for words; *d’* = 1.56, 0.84, −0.08, −0.18, −0.36, and −0.47 for pseudowords), and also at a “fast” speech rate (*d’* = 2.26, 1.82, 1.30, 0.84, 0.54, and 0.65 for words; *d’* = −0.02, −0.87, −0.80, −0.67, −0.44, and −0.24 for pseudowords). Stop-dominant *words* remained intelligible across all six levels of speech degradation, regardless of speech rate, with *d’* values above 0.5 (approximately chance-level intelligibility). On the other hand, *pseudowords* were only intelligible when spoken slowly and with the reversed segment length up to 30 ms. The intelligibility of words was increasingly supported by slower speech rates as degradation increased in six steps, while pseudowords showed the opposite trend.

An ANOVA was performed with speech rate (fast vs. slow), lexicality (words vs. non-words), and reversed segment length (10–110 ms) as within-subjects factors. The results showed that locally time-reversed items spoken at a “slow” speech rate were significantly more intelligible than those spoken at a “fast” speech rate, *F*(1, 29) = 155.49, *p* < 0.001, partial η^2^ = 0.84. Additionally, words were significantly more intelligible than pseudowords when locally time-reversed, *F*(1, 29) = 184.00, *p* < 0.001, partial η^2^ = 0.86. The intelligibility of locally time-reversed items deteriorated significantly with increasing reversed segment length, *F*(5, 145) = 80.44, *p* < 0.001, partial η^2^ = 0.74. There was also a significant interaction between speech rate and reversed segment length, *F*(5, 145) = 4.51, *p* = 0.001, partial η^2^ = 0.14, indicating that the intelligibility degradation patterns with increasing reversed segment length differed significantly between “fast” and “slow” speech. A significant interaction was also found between lexicality and reversed segment length, *F*(5, 145) = 7.43, *p* < 0.001, partial η^2^ = 0.20, indicating that the intelligibility degradation patterns with increasing reversed segment length differed significantly between words and pseudowords. On the other hand, there was *no* significant interaction between speech rate and lexicality, *F*(1, 29) = 3.39, *p* = 0.076, partial η^2^ = 0.11, indicating that the intelligibility gap between words and pseudowords was not significantly different between “fast” and “slow” speech. Overall, a significant three-way interaction was found between speech rate, lexicality, and reversed segment length, *F*(5, 145) = 28.99, *p* < 0.001, partial η^2^ = 0.50, indicating that the intelligibility gap between words and pseudowords with increasing reversed segment length, differed significantly between “fast” and “slow” speech.

Additionally, to examine the effects of the “slow” speech rate, compared to the “normal” speech rate (Ishida, 2021), on the intelligibility of locally time-reversed words and pseudowords, an ANOVA was performed with speech rate (slow vs. normal) as a between-subjects factor, and lexicality (words vs. pseudowords) and reversed segment length (10–110 ms) as within-subjects factors. The results showed that locally time-reversed items spoken at a “slow” speech rate were significantly more intelligible than those spoken at a “normal” speech rate, *F*(1, 58) = 4.87, *p* = 0.031, partial η^2^ = 0.08. Additionally, words were significantly more intelligible than pseudowords when locally time-reversed, *F*(1, 58) = 329.03, *p* < 0.001, partial η^2^ = 0.85. Furthermore, the intelligibility of locally time-reversed items deteriorated significantly with increasing reversed segment length, *F*(4.69, 271.85) = 134.23, *p* < 0.001, partial η^2^ = 0.70 (Huynh–Feldt correction). There was also a significant interaction between lexicality and reversed segment length, *F*(4.42, 256.50) = 28.48, *p* < 0.001, partial η^2^ = 0.33 (Huynh–Feldt correction), indicating that the intelligibility degradation patterns with increasing reversed segment length differed significantly between words and pseudowords. On the other hand, there was *no* significant interaction between lexicality and speech rate, *F*(1, 58) = 1.10, *p* = 0.299, partial η^2^ = 0.02, indicating that the intelligibility gap between words and pseudowords was *no*t significantly different between “slow” and “normal” speech. There was also *no* significant interaction between speech rate and the reversed segment length, *F*(4.69, 271.85) = 2.20, *p* = 0.058, partial η^2^ = 0.04 (Huynh–Feldt correction), indicating that the intelligibility degradation patterns with increasing reversed segment length were not significantly different between “slow” and “normal” speech. Overall, *no* significant interaction was found between speech rate, lexicality, and reversed segment length, *F*(4.42, 256.50) = 2.11, *p* = 0.073, partial η^2^ = 0.04 (Huynh–Feldt correction), indicating that the gap between words and pseudowords with increasing reversed segment length, was not significantly different between “slow” and “normal” speech.

Furthermore, to examine the effects of the “fast” speech rate, compared to the “normal” speech rate (Ishida, 2021), on the intelligibility of locally time-reversed words and pseudowords, another ANOVA was performed with speech rate (fast vs. normal) as a between-subjects factor, and lexicality (words vs. pseudowords) and reversed segment length (10–110 ms) as within-subjects factors. The results showed that locally time-reversed items spoken at a “fast” speech rate were significantly less intelligible than those spoken at a “normal” speech rate, *F*(1, 58) = 74.10, *p* < 0.001, partial η^2^ = 0.56. Additionally, words were significantly more intelligible than pseudowords when locally time-reversed, *F*(1, 58) = 261.03, *p* < 0.001, partial η^2^ = 0.82. The intelligibility of locally time-reversed items deteriorated significantly with increasing reversed segment length, *F*(5, 290) = 96.57, *p* < 0.001, partial η^2^ = 0.63. There was a significant interaction between lexicality and reversed segment length, *F*(4.50, 260.96) = 11.92, *p* < 0.001, partial η^2^ = 0.17 (Huynh–Feldt correction), indicating that the intelligibility degradation patterns with increasing reversed segment length differed significantly between words and pseudowords. Moreover, there was a significant interaction between lexicality and speech rate, *F*(1, 58) = 5.29, *p* = 0.025, partial η^2^ = 0.08, indicating that the intelligibility gap between words and pseudowords differed significantly between “fast” and “normal” speech. There was also a significant interaction between speech rate and reversed segment length, *F*(5, 290) = 6.81, *p* < 0.001, partial η^2^ = 0.11, indicating that the intelligibility degradation patterns with increasing reversed segment length differed significantly between “fast” and “normal” speech. Overall, there was a significant three-way interaction between speech rate, lexicality, and reversed segment length, *F*(4.50, 260.96) = 25.97, *p* < 0.001, partial η^2^ = 0.31 (Huynh–Feldt correction), indicating that the intelligibility gap between words and pseudowords with increasing reversed segment length, differed significantly between “fast” and “normal” speech.

Overall, stop-dominant Japanese words were significantly more intelligible than pseudowords when locally time-reversed, regardless of speech rate, highlighting robust lexical support in perceptual restoration. Additionally, locally time-reversed items spoken at a “slow” speech rate were significantly more intelligible than those spoken at a “fast” speech rate. However, when comparing “slow” and “normal” speech rate, the advantage of slower speech rate was less evident. While the “slow” speech rate improved intelligibility overall (main effect), the lexical advantage (words > pseudowords) observed in perceptual restoration across six levels of speech degradation, did not differ significantly between “slow” and “normal” speech (no three-way interactions). Conversely, “fast” speech was particularly vulnerable to local time distortions, resulting in significantly reduced intelligibility compared to “normal” speech, though lexicality continued to support perceptual restoration with increasing reversed segment lengths. In conclusion, stop-dominant Japanese words were consistently more intelligible than pseudowords, and the “slow” speech rate in speech greatly facilitated perceptual restoration compared to the “fast” speech rate.

## General discussion

The current study examined the robustness of locally time-reversed Japanese words and pseudowords by imposing more severe temporal distortions than those used in previous research at the lexical level. It focused on the perceptual restoration mechanisms in Japanese – a language that has not been fully explored in past studies. Specifically, this study explored potential language-specific robustness in Japanese, compared to English, reflecting the fact that Japanese has a consecutive CV (consonant–vowel) structure and tends to retain high intelligibility under local time reversal (Ishida, 2021). Two distinct types of severe temporal distortion were tested: long reversed segment lengths exceeding 100 ms and altered speech rates (fast and slow).

In Experiment 1, native Japanese speakers listened to locally time-reversed Japanese words and pseudowords with reversed segment lengths of 100, 120, 140, 160, 180, and 200 ms, which had not been examined previously. The results showed the strong robustness of Japanese words and pseudowords to temporal distortions, with a strong influence of dominant-phoneme type (fricative > stop) and lexicality (words > pseudowords) in perceptual restoration. The advantage of dominant-phoneme type (fricative > stop) was also more evident under severe temporal distortion (100–200 ms), where fricative-dominant items (both words and pseudowords) were significantly more intelligible than stop-dominant items; compared to previous studies where *stop-dominant words* were more intelligible than *fricative-dominant pseudowords* at shorter reversed segment lengths (10–110 ms) (Figs. 2 and 3). These findings suggest acoustic/phonetic properties play a stronger role in perceptual restoration under severe speech degradation, while lexicality consistently supports the process. Overall, Japanese words and pseudowords demonstrated greater tolerance to severe temporal distortion compared to their English counterparts in previous research (Ishida et al., 2016).

Experiment 2 further examined the intelligibility of stop-dominant Japanese words and pseudowords, which were vulnerable to local time distortion, by naturally altering the speech rate to either “fast” or “slow” and imposing local time reversal at intervals of 10, 30, 50, 70, 90, and 110 ms (the segment lengths commonly used in our previous studies with a “normal” speech rate). The results showed that words were significantly more intelligible than pseudowords, and the “slow” speech rate significantly enhanced perceptual restoration compared to the “fast” speech rate. In other words, the fast speech rate was highly vulnerable to local time distortions, leaving pseudowords with little chance of being understood.

Additionally, when comparing the results of Experiment 2 with past research (Fig. 4), *stop-dominant Japanese words* spoken at a slow or normal speech rate were generally more intelligible than stop-dominant English words spoken at a normal speech rate (words: Japanese > English). However, *stop-dominant Japanese pseudowords* spoken at a slow or normal speech rate were generally less intelligible than stop-dominant English pseudowords spoken at a normal speech rate (pseudowords: English > Japanese). While Experiment 1 demonstrated the general robustness of Japanese words and pseudowords under severe temporal distortion, Experiment 2 revealed that the robustness under varying speech rates was evident for Japanese words but not for Japanese pseudowords. This difference may be attributed to the basic linguistic structure of Japanese and its effects on perceptual restoration.

That is, Japanese is a mora-based language characterized by the basic mora structure of CV (consonant–vowel) (Table 1). When a target word consists of CVCVCVCV with four morae, and when a word and its matched pseudoword differ by only one consonant, listeners’ perception and perceptual restoration can be strongly influenced by their prior knowledge, possibly leading them to perceive the target word under acoustic degradation as a real word. The regular occurrence of vowels also provides strong lexical context, making it easier for listeners to perceive a familiar word. Even when listeners are exposed to pseudowords that differ by only one phoneme, they may still perceive a real word by perceptually restoring degraded speech. In other words, Japanese listeners may be more influenced by acoustic and lexical context, leading them to perceive an existing word, even when the target is a pseudoword. In contrast, speakers of syllable-based languages like English may rely more on varied consonant clusters in syllables, leading them to accurately perceive sounds and recognize pseudowords, rather than perceptually restoring degraded speech based primarily on acoustic and/or lexical context. Even when the average number of morae and syllables in the target speech is equivalent, and even when a word and its pseudoword differ by only one consonant, perception can vary depending on whether the consonant change occurs within the CV mora structure or within the syllable structure of various consonant–vowel combinations. In other words, the basic linguistic structure can influence how degraded speech is perceived and restored.

Comparing the basic linguistic structure in Japanese (mora) and English (syllable), the number of basic linguistic structure in Japanese is very limited. Japanese has CV (consonant–vowel) and V (vowel;/i/,/e/,/a/,/o/, or/u/only) as basic sound units, along with some special morae such as of C and CCV. Among these, the CV structure is the most frequent sound unit in Japanese. In this structure, a single consonant is typically adjacent to a vowel or surrounded by vowels. In contrast, English has a greater variety of syllables as basic sound units such as V, VC, VCC, VCCC, CV, CVC, CVCC, CVCCC, CVCCCC, CCV, CCVC, CCVCC, CCVCCC, CCVCCCC, CCCV, CCCVC, CCCVCC, CCCVCCC (Bergman et al., 2007; Celce-Murcia et al., 2010; Kono, 2004), often characterized by the frequent occurrence of consonant clusters. The frequent occurrence of oral vowels and the rare presence of consonant clusters in Japanese might have contributed to the high intelligibility of Japanese words and low detectability of pseudowords (due to perceptual restoration) under the severe temporal distortions.

In fact, vowels are very tolerant of temporal distortions. Pellegrino et al. (2010) reported that oral vowels in French were identified with 88.6% accuracy when French pseudowords consisting of CVC were globally inverted (i.e., played backward) – vowels were intelligible even when reversed in time. Drullman et al. (1994) also reported that vowels were more intelligible than consonants when Dutch pseudowords (CVC and VCV) were temporally and acoustically smeared. In their study, speech signals were divided into ¼ octave bands, and the amplitude envelope of each band was low-pass filtered with cut-off frequencies of 16 Hz, 8 Hz, 4 Hz, 2 Hz, and 0 Hz. In other words, the modulation frequency components of the speech signal were low-pass filtered, resulting in changes to the amplitude envelope of speech: the lower the cut-off frequency, the flatter the envelope and less rapid the spectral changes. While intelligibility deteriorated as lower cut-off frequencies were applied in five steps, vowels retained 56% intelligibility at a low-pass cut-off frequency of 0 Hz, while consonants retained less than half of their intelligibility under this most severe temporal distortion (with the identification target being the initial consonant, vowel, final consonant for CVC and the consonant in the middle for VCV). Speech with a frequent occurrence of vowels, such as Japanese CVCVCVCV words, would therefore possibly maintain high intelligibility under severe temporal distortion, providing both acoustic and linguistic cues for perceptual restoration.

Additionally, some consonants are more tolerant of acoustic distortion than others. Pellegrino et al. (2010) reported that French consonants in globally time-reversed CVC pseudowords were perceptually restored with varying accuracy rates depending on the broad phonetic class: 95.8% for liquids, 93.3% for voiceless fricative, 91.7% for voiced fricatives, 90.0% for nasals, 66.7% for rhotics, 61.8% for voiced stops, and 9.4% for voiceless stops. Fricatives were generally perceived with high accuracy (over 90%), while stops were perceived with the least accuracy. This difference may be attributed to the acoustic features of fricatives, which tend to have relatively symmetric temporal envelopes, whereas stops are more asymmetric due to the presence of voice onset time (VOT). Drullman et al. (1994) also reported that Dutch consonants in CVC and VCV pseudowords under acoustic smearing (where the modulation frequency components were low-pass filtered with cut-off frequencies of 8 Hz, 4 Hz, 2 Hz, or 0 Hz) were more accurately restored when the consonants were either fricatives (/f, s, χ, v, z/) or vowel-like consonants (/m, n, ŋ, l, w, j, h/), while stops (/t, k, p, b, d/) were least accurately restored due to their short duration. When the temporal envelope was smeared by temporal reversal or modulation filtering, consonants that are relatively long in duration, high in amplitude, and symmetric in the structure tended to retain their intelligibility better than others.

Considering that both vowels and certain consonants are highly tolerant of acoustic distortion, it is reasonable to assume that speech is more intelligible when it contains these robust phonemes. Japanese, with CV as its basic sound unit, would also exhibit high intelligibility when the two phonemes in CV are acoustically robust. The regular occurrence of a vowel and a single consonant in the Japanese CV structure (as in CVCVCVCV) likely contributes to high intelligibility under severe temporal distortion. In general, the robustness of speech under degradation is facilitated by acoustic similarity between the original and manipulated signal, often referred to as the “masking potential rule” (Bashford & Warren, 1987; Bashford et al., 1992; Houtgast, 1972; Jakobson et al., 1952; Kashino, 2006; Kashino & Warren, 1996; Warren et al., 1972). For example, a deleted sound can be perceptually restored when replaced by a sound acoustically similar to the original (Samuel, 1981a, 1981b; Warren, 1970; Warren & Warren, 1970). Missing sounds are restored more effectively when the replacing sound is “as loud as or louder than the loudest sound in the sentence” (Warren & Warren, 1970) and has the same center frequency as the original sound (Bashford & Warren, 1987; Warren, 1972; Warren et al., 1972). The degree of restorability depends on acoustic similarity (Samuel, 1981a, 1981b) – stops and fricatives are most successfully restored when replaced by white noise, while nasals and liquids are restored to a moderate extent, and vowels are least restored. Interestingly, these patterns reverse when the replacing sound is a pure tone: vowels are then restored most effectively, followed by nasals and liquids, and finally stops and fricatives). Additionally, degraded speech is restored more effectively when the original amplitude envelope is better preserved (Ishida, 2021; Ishida & Arai, 2016; Ishida et al., 2016; Ishida et al., 2018; Stevens, 1999). In locally time-reversed speech, the temporal components of the speech signal are shifted forward or backward from their original temporal positions, while all the original sound elements remain. The temporal envelope of speech also gradually alters as the reversed segment lengths become longer (Ishida et al., 2018). The robustness of Japanese words and pseudowords under local time reversal is presumably supported by acoustic similarity between the speech signal before and after the reversal (where the original sound component was replaced by another component from the same speech), reinforced by the acoustic characteristics of the CV structure, and reflected in the degree of preservation of the original temporal envelope.

Slower speech also facilitates perceptual restoration more effectively than fast speech, as it provides clearer articulation and longer durations. In contrast, fast speech tends to have lower intelligibility due to overlapping articulatory motions, which obscure the acoustic and linguistic cues listeners expect in speech perception (Brown & Hilferty, 1982, 1986, 1995, Brown & Kondo-Brown, 2006; Dalby, 1986;; Johnson, 2004; Stilp et al., 2010). Perceptual restoration is most successful when acoustic features (e.g., spectral change and transitions) align with listeners’ expectations, aided by an appropriate speech rate and clearer articulation (Grataloup et al., 2009; Kashino, 2006; Kiss et al., 2008; Warren & Obusek, 1971; Warren & Sherman, 1974; Warren & Warren, 1970). Degraded acoustic signals can be effectively decoded into language when sufficient acoustic and linguistic information is available and successfully combined for speech perception (Best, 1994; Bond, 1999; Bregman, 1990; Cherry, 1953; Cherry & Wiley, 1967; Cutler, 1997; Dalby, 1986; Denes & Pinson, 1963; Johnson, 2004; Liberman, et al., 1952; Liberman, et al., 1967; Luce et al., 1998; Massaro, 1987; Pisoni, 1978; Voss, 1984). Japanese speech, characterized by the regular alternation of consonants and vowels (CV), can reasonably be assumed to be highly tolerant of temporal distortion and, consequently, more intelligible – especially when spoken clearly at a slower rate – thanks to its acoustically robust linguistic structure and the predictability of words in consecutive CV contexts.

In summary, the current study examined the robustness of Japanese words and pseudowords under severe temporal distortion – a condition not previously explored at the lexical level. Experiment 1 investigated the intelligibility of Japanese words and pseudowords containing either many fricatives or stops, subjected to heavy temporal reversals at intervals of 100–200 ms. The results showed high intelligibility, supported by dominant phoneme type (fricative > stop) and lexicality (words > pseudowords). Experiment 2 further examined stop-dominant words and pseudowords (which were more susceptible to temporal distortions in Experiment 1) at fast or slow rates with local time reversals of 10–110 ms. The results showed strong effects of lexicality (words > pseudowords), along with speech rate (slow > fast). While lexicality consistently facilitated perceptual restoration, the effects of dominant phoneme type (Experiment 1) and speech rate (Experiment 2) were prominent under severe temporal inversion. Comparing the current results with past research on degraded speech in different languages, the high intelligibility of Japanese degraded speech appears to be strongly facilitated by its CV structure, where a vowel (robust to acoustic degradation) follows a consonant, which may also be acoustically tolerant depending on the phoneme class. The current study tentatively concludes that Japanese words are highly tolerant of severe temporal distortions, with the CV structure of Japanese supporting perceptual restoration.

## Appendix A

**Table:** The list of fricative-dominant and stop-dominant words and pseudowords for Experiment 1 and 2, adopted from Ishida (2021). The words were originally selected from Amano and Kondo (2000)

Fricative-dominant words	Fricative-dominant pseudowords	Stop-dominant words	Stop-dominant pseudowords
hishigata	hi**h**igata	bakuyaku	ba**p**uyaku
hikidashi	hikida**h**i	butaniku	bu**p**aniku
hitosama	hito**h**ama	datemaki	da**p**emaki
hitohada	hito**s**ada	dekasegi	de**p**asegi
hiyakashi	hiyaka**h**i	dekigoto	de**p**igoto
hirusugi	hiru**f**ugi	dekitate	deki**p**ate
hirumeshi	hirume**h**i	dokudami	doku**b**ami
hahakata	ha**s**akata	dokugaku	doku**d**aku
hahanohi	ha**s**anohi	dokuhebi	do**p**uhebi
hakanasa	hakana**h**a	gakubuchi	ga**p**ubuchi
hagishiri	hagi**h**iri	gakureki	ga**p**ureki
hakidasu	hakida**f**u	garakuta	gara**p**uta
hakuhatsu	haku**s**atsu	gekitotsu	ge**p**itotsu
hageshisa	hage**h**isa	getsugaku	getsu**d**aku
hachinosu	hachino**f**u	gobusata	go**g**usata
hanashite	hana**h**ite	gokubuto	go**p**ubuto
hanafuda	hana**s**uda	gokuraku	go**p**uraku
harusaki	haru**h**aki	kabekake	kabe**p**ake
harusame	haru**h**ame	kakesoba	ka**p**esoba
hesokuri	he**h**okuri	kakutoku	ka**p**utoku
hoshigaki	ho**h**igaki	kamaboko	kamabo**t**o
hoshigaru	ho**h**igaru	kamigata	kami**d**ata
hoshikusa	ho**h**ikusa	katagaki	ka**p**agaki
hosomeru	ho**h**omeru	katakana	kata**p**ana
horidasu	horida**f**u	katakoto	kata**p**oto
horobosu	horobo**f**u	kigakari	kiga**p**ari
fushimatsu	fu**h**imatsu	kigokochi	kigo**p**ochi
fukashigi	fuka**h**igi	kikikata	ki**p**ikata
fukisoku	fuki**h**oku	kikitori	kiki**p**ori
fukidasu	fukida**f**u	kikubari	kiku**d**ari

Fricative-dominant words	Fricative-dominant pseudowords	Stop-dominant words	Stop-dominant pseudowords
fuyufuku	fuyu**s**uku	kirikabu	kiri**p**abu
furisode	furi**h**ode	kobutori	ko**g**utori
furidashi	furida**h**i	kodakara	ko**g**akara
furusato	furu**h**ato	kokuhaku	ko**p**uhaku
furoshiki	furo**h**iki	kokuseki	ko**p**useki
sasageru	sa**h**ageru	kotobuki	koto**g**uki
sashihiki	sashi**sh**iki	kotogara	ko**p**ogara
sakaseru	saka**h**eru	kuchibeta	kuchi**g**eta
sakihodo	saki**s**odo	kugiduke	ku**b**iduke
sakeguse	sakegu**h**e	kujibiki	kuji**g**iki
sabishige	sabi**h**ige	kumitate	kumi**p**ate
seseragi	se**h**eragi	kitaguni	ki**p**aguni
sekisetsu	seki**h**etsu	kuzukago	kuzu**p**ago
setsunasa	setsuna**h**a	tabibito	tabi**g**ito
shizukesa	shizuke**h**a	takatobi	ta**p**atobi
shitashisa	shita**h**isa	takenokoto	ta**p**enokoto
shitsukosa	shitsuko**h**a	takoyaki	ta**p**oyaki
shibutosa	shibuto**h**a	tanabata	tana**g**ata
shimedasu	shimeda**f**u	tategaki	tate**b**aki
shiraseru	shira**h**eru	tebukuro	te**g**ukuro
shiromiso	shiromi**h**o	tedasuke	te**b**asuke
shiwayose	shiwayo**h**e	tekagami	teka**b**ami
susumeru	su**f**umeru	tekikaku	teki**p**aku
suzushige	suzu**h**ige	tetsugaku	tetsu**d**aku
suzumushi	suzumu**h**i	tobibako	tobi**d**ako
sunahama	suna**s**ama	tobikomi	to**g**ikomi
subayasa	subaya**h**a	tokubetsu	toku**g**etsu
surudosa	surudo**h**a	tokuyaku	to**p**uyaku
sokonashi	sokona**h**i	tonogata	tono**b**ata
sobakasu	sobaka**f**u	toritate	tori**p**ate

## Declarations

### Open practices statement

The data for all experiments are available via the Open Science Framework at: https://osf.io/93yvg/?view_only=01c70520ad64468f9ad2e80de19305b2

Any further information is available upon request from the corresponding author. This study was not preregistered.

### Open practices statement

The data for all experiments are available via the Open Science Framework at: https://osf.io/93yvg/?view_only=01c70520ad64468f9ad2e80de19305b2. Any further information is available upon request from the corresponding author. This study was not preregistered.
